# A Nanopore Based Chromosome-Level Assembly Representing Atlantic Cod from the Celtic Sea

**DOI:** 10.1534/g3.120.401423

**Published:** 2020-07-08

**Authors:** Tina Graceline Kirubakaran, Øivind Andersen, Michel Moser, Mariann Árnyasi, Philip McGinnity, Sigbjørn Lien, Matthew Kent

**Affiliations:** * Centre for Integrative Genetics and Department of Animal and Aquacultural Sciences, Faculty of Biosciences, Norwegian University of Life Sciences, Ås, Norway; † Nofima, Ås, Norway; ‡ School of Biological, Earth & Environmental Sciences, University College Cork, Ireland

**Keywords:** Atlantic cod, genome assembly, nanopore, chromosomal rearrangements, linkage map, centromere repeats

## Abstract

Currently available genome assemblies for Atlantic cod (*Gadus morhua*) have been constructed from fish belonging to the Northeast Arctic Cod (NEAC) population; a migratory population feeding in the Barents Sea. These assemblies have been crucial for the development of genetic markers which have been used to study population differentiation and adaptive evolution in Atlantic cod, pinpointing four discrete islands of genomic divergence located on linkage groups 1, 2, 7 and 12. In this paper, we present a high-quality reference genome from a male Atlantic cod representing a southern population inhabiting the Celtic sea. The genome assembly (gadMor_Celtic) was produced from long-read nanopore data and has a combined contig length of 686 Mb with an N50 of 10 Mb. Integrating contigs with genetic linkage mapping information enabled us to construct 23 chromosome sequences which mapped with high confidence to the latest NEAC population assembly (gadMor3) and allowed us to characterize, to an extent not previously reported large chromosomal inversions on linkage groups 1, 2, 7 and 12. In most cases, inversion breakpoints could be located within single nanopore contigs. Our results suggest the presence of inversions in Celtic cod on linkage groups 6, 11 and 21, although these remain to be confirmed. Further, we identified a specific repetitive element that is relatively enriched at predicted centromeric regions. Our gadMor_Celtic assembly provides a resource representing a ‘southern’ cod population which is complementary to the existing ‘northern’ population based genome assemblies and represents the first step toward developing pan-genomic resources for Atlantic cod.

Atlantic cod (*Gadus morhua*) is a commercially exploited high-fecundity fish with a wide geographical distribution extending over the North Atlantic Ocean from the nearly freezing waters in the Arctic to variable high temperatures typical of the southern extremities of the species’ Eastern Atlantic distribution (Mieszkowska *et al.* 2009; Righton *et al.* 2010; Morris *et al.* 2018). It has been proposed that increases in water temperatures associated with global warming will see Atlantic cod spread northwards and occupy larger areas of Barents Sea, while southern populations will decline and possibly disappear (Drinkwater 2005; Mieszkowska *et al.* 2009). Characterizing the genomic diversity among fish populations, and understanding its relationship to phenotypic variation has become increasingly important in fisheries management and for predicting the response of various ecotypes to environmental fluctuations, such as climatic changes (Neat and Righton 2007; Righton *et al.* 2010). An early genetic map from (Hubert *et al.* 2010) presented a standardized nomenclature of cod linkage groups (which we have followed in our work), and which has been used in multiple studies providing evidence for elevated genomic divergence among populations mainly in respect of four discrete genomic regions, also referred as supergenes, located on linkage groups (LGs) 1, 2, 7 and 12 (Hemmer-Hansen *et al.* 2013; Karlsen *et al.* 2013; Berg *et al.* 2015; Berg *et al.* 2016; Kirubakaran *et al.* 2016; Sodeland *et al.* 2016; Barney *et al.* 2017; Berg *et al.* 2017; Barth *et al.* 2019; Kess *et al.* 2019). Relationships between these regions and environmental conditions indicates that the region identified on LG01 is associated with strong genetic differentiation between migratory and stationary ecotypes on both sides of the Atlantic Ocean (Hemmer-Hansen *et al.* 2013; Karlsen *et al.* 2013; Berg *et al.* 2016; Kirubakaran *et al.* 2016; Sinclair-Waters *et al.* 2017; Kess *et al.* 2019). This supergene coincides with a double inversion that suppresses homologous recombination in heterozygotes and effectively prevents admixing between co-segregating haplotypes (Kirubakaran *et al.* 2016). The genomic islands of divergence on LGs 2, 7 and 12 are also found on both sides of the Atlantic Ocean and it has been suggested that they are associated with mean ocean temperatures along the north-south gradient (Bradbury *et al.* 2010; Bradbury *et al.* 2013; Berg *et al.* 2015; Clucas *et al.* 2019). Genomic divergence in these regions has also been associated with other environmental factors in studies comparing Baltic and North Sea populations (Berg *et al.* 2015), as well as oceanic and coastal populations in the North Sea (Sodeland *et al.* 2016). Elevated linkage disequilibrium (LD) detected across the regions on LGs 2, 7, and 12 are likely to have arisen as a result of chromosomal inversions, but high-resolution sequence data showing this and describing the precise locations, sizes and genomic structure underlying these regions has so far been lacking.

Most fish genome sequences have been built from short-read Illumina data, which is a computationally challenging and error prone process especially when the genomes contain extensive repetitive regions. Long-read sequencing technologies provide the means to directly read through repetitive elements and thereby potentially produce much more complete *de novo* assemblies. The recently released gadMor3 assembly (NCBI accession ID: GCF_902167405.1) was developed based on long-read sequence data produced from a NEAC fish and represent a significant improvement over previous gadMor1 and gadMor2 assemblies generated from the same northern population (Star *et al.* 2011; Tørresen *et al.* 2017). In this paper, we used long-read nanopore data to construct a reference genome assembly for a male Atlantic cod from the southern population of the Celtic Sea and integrated the assembly with linkage data to build high-quality chromosomes sequences. The genome sequence was utilized to detect a potential centromeric repeat sequence differentiating chromosomal morphology and to characterize with high precision the chromosomal rearrangements underlying the notable supergenes on LGs 1, 2, 7 and 12.

## Materials and Methods

### Sample, DNA extraction and sequencing

DNA from a single, male cod (45cm, 1009g) fished in the Celtic Sea in January (50° 42.16N 07° 53.27W, 110m depth) was extracted from frozen blood using the Nanobind CBB Big DNA kit from Circulomics and sequenced using a PromethION instrument from Oxford Nanopore Technology (ONT). Using 1.5ug DNA and starting material, two sequencing libraries were generated following the ligation protocol (SQK-LSK109, ONT), one using DNA fragments >20kb, size selected using a BUF7510 High pass cassette run on a Blue Pippin (Sage Scientific), and another where no size selection was performed. Both libraries were split in two and each half sequenced successively on the same flow-cell (type R9.4.1) after nuclease flushing according to the Oxford Nanopore protocol (version: NFL_9076_v109_revF_08Oct2018). Combined data yields after quality filtering were 11.2 and 35.5 billion bases for size selected and non-size selected respectively, with median read lengths being 23.3 kb and 4.5 kb. Together this represents approximately 70X long-read genome coverage assuming an Atlantic cod genome size of 670 Mb (as is reported for the gadMor3 assembly; GenBank accession GCA_902167405.1). Short read data (2 × 250bp) was generated from non-size selected DNA using an Illumina MiSeq instrument. Libraries were prepared using a TruSeq DNA PCR free kit (Illumina) and sequenced in multiple runs to generate 71M read pairs, equalling approximately 35.5Gbp or 50X genome coverage.

### Construction of the gadMor_Celtic assembly

The raw nanopore reads (n = 2,868,527) were base-called using Guppy-2.2.3 (https://community.nanoporetech.com) using the flip-flop model. Adapters were removed from reads using Porechop v0.2.3, 1 (Wick *et al.* 2018) and quality-filtered using fastp v.0.19.5.2 (Chen *et al.* 2018) with mean base quality greater than 7, trimming the 50bp at the 5′ end of the read and removing all reads less than 4000 bp. Multiple initial assemblies applying various parameters were produced using wtdgb2 v2.3 (Ruan and Li 2020) (File S1). Based on relative values for number of contigs, contig N50 and total genome size, the completeness of selected assembled genomes was estimated using Benchmarking Universal Single-Copy Orthologs (BUSCO) v3.1.0 (Simão *et al.* 2015) and applying the actinopterygii (ray-finned fishes) reference gene data set. Two genome assemblies (“trial c”; *P* = 23, k = 0, L = 10000, e = 3, A = 4, S = 4, s = 0.05, and “trial q”; *P* = 23, k = 0, L = 10000, e = 3, A = 3, S = 3, s = 0.05; See file S1 for additional details) with the overall relative best assembly and BUSCO scores were selected for further processing.

To improve assembly contiguity, contigs showing a sequence overlap of more than 5000bp and similarity >95% were combined using quickmerge (Chakraborty *et al.* 2016). This consensus assembly was error corrected by performing two successive rounds of processing by Racon v2.3 (Vaser *et al.* 2017) using only quality filtered nanopore reads. Raw MiSeq reads were quality filtered using Trimmomatic v0.32, before being used by Pilon v1.23 to further improve per-base accuracy in the consensus sequence. Completeness of the final polished contigs was performed as described above using BUSCO. All parameters used for processing of nanopore reads and generating genome assemblies is described in File S1.

### Linkage mapping and construction of chromosome sequences

The linkage map was constructed using data from 9,178 polymorphic SNPs (Table S1) displaying >95% genotype call rates in farmed cod (n = 2951) sampled from 88 full-sib families of the National cod breeding program maintained by Nofima in Tromsø, Norway, and from eight full-sib families of the CODBIOBANK at the Institute of Marine Research in Bergen, Norway. The genotypes were generated using a SNP-array created as a part of the Cod SNP Consortium (CSC) in Norway, and which has been used in numerous previous studies (Berg *et al.* 2015; Sodeland *et al.* 2016; Barth *et al.* 2017; Berg *et al.* 2017; Sinclair-Waters *et al.* 2017; Knutsen *et al.* 2018; Kess *et al.* 2019). The SNPs included on this array were selected to be evenly distributed across larger gadMor1 assembly contigs and are expected to be a good foundation for anchoring sequences to chromosomes. Linkage mapping was performed with the Lep-MAP (V2.0) software in a stepwise procedure (Rastas *et al.* 2013). First, SNPs were assigned to linkage groups with the ‘SeparateChromosomes’ command using increasing LOD thresholds until the observed number of linkage groups corresponded with the expected haploid chromosome number of 23 (Ghigliotti *et al.* 2012). Additional SNPs were subsequently added to the groups with the ‘JoinSingles’ command at a more relaxed LOD threshold, and finally SNPs were ordered in each linkage group with the ‘OrderMarkers’ command. Following this, sequence flanking each marker was used to precisely position all genetic markers to contigs in the gadMor_Celtic assembly using megablast (Altschul *et al.* 1990), and thereby associate sequence with linkage groups. This analysis revealed 2 chimeric contigs containing at markers from each of different linkage groups that were selectively ‘broken’ using alignments with the gadMor2 assembly (Tørresen *et al.* 2017). After breakage of the two contigs, linkage information was used to order, orientate and concatenate contigs into 23 sequence files. Finally, SNPs were positioned in the sequences using megablast and linkage maps constructed using a fixed order in Lep-MAP to produce the final male and female linkage maps presented in Table S1. The gadMor_Celtic assembly was aligned against the recently released gadMor3 assembly (NCBI accession ID: GCF_902167405.1) using LASTZ (Harris 2007); see S1 for details.

### Detection of repetitive elements

RepeatModeler version 1.0.8 (Smit *et al.* 1996) was used to generate a repeat library, subsequently RepeatMasker version 4.0.5 (Smit *et al.* 1996) was run on the finished gadMor_Celtic with default options to identify the repeats in the genome assembly. For the purposes of detecting putative centromeric sequences, tandem repeats were identified using TandemRepeat finder (TRF) version 4.09 (Benson 1999). The output was processed using custom perl and unix scripts to identify repeats specifically containing more than 60% AT, longer than 80 bp, and present in all 23 LGs.

### Gene annotation

Data from various public sources was used to build gene models including (i) 3M transcriptome reads generated using GS-FLX 454 technology and hosted at NCBI’s SRA (https://www.ncbi.nlm.nih.gov/sra/?termc=cSRP013269), (ii) >250K ESTs hosted by NCBI (https://www.ncbi.nlm.nih.gov/nucest) (iii) 4.4M paired-end mRNA MiSeq sequences from whole NEAC larvae at 12 and 35 dph (https://www.ebi.ac.uk/ena, PRJEB25591) and (iv) 362M Illumina reads from 1 and 7 dph (https://www.ebi.ac.uk/ena, PRJEB25591). To enable model building, MiSeq reads and short illumina reads were mapped to the gadMor_Celtic assembly using STAR v2.3.1z (Dobin *et al.* 2013), while 454 transcriptome reads were mapped using gmap v2014-07-28 (Wu and Watanabe 2005) with ‘–no-chimeras’ parameter in addition to default parameters. stringtie v1.3.3 (Pertea *et al.* 2015) was used to assemble the reads into transcript models. Transcript models were merged using stringtie merge (Pertea *et al.* 2015). Gene models were tested by performing (i) open reading frame (ORF) prediction using TransDecoder (Haas *et al.* 2013) using both pfamA and pfamB databases for homology searches and a minimum length of 30 amino acids for ORFs without pfam support, and (ii) BLASTP analysis (evalue <1e-10) for all predicted proteins against zebrafish (*Danio rerio*) (v9.75) and three-spined stickleback (*Gasterosteus aculeatus*) (BROADS1.75) annotations from Ensembl. Only gene models with support from at least one of these homology searches were retained. Functional annotation of the predicted transcripts was done using blastx against the SwissProt database. Results from TransDecoder and homology support filtering of putative protein coding loci are shown in Table S2.

### Data availability

The datasets generated and used during the current study, the gadMor_Celtic assembly, all supplementary files, and the repeat library are available at figshare: doi.org/10.6084/m9.figshare.10252919. The genome assembly gadMor_Celtic is available in NCBI under the genbank accession id GCA_010882105.1. The custom script is available in https://github.com/GracelinTina/gadMor_Celtic. The raw nanopore reads and illumina MiSeq reads used to generate gadMor_Celtic are available at European Nucleotide Archive under accession ID PRJEB35290.

## Results and Discussion

### Genome assembly

The current methodological convention in population genomics is to build genomic tools and interpret results based on the information acquired from one arbitrarily sampled individuals’ reference genome, which is used as a default to represent the whole species. Accordingly, genome assemblies for Atlantic cod have been generated from NEAC, which is a migratory population feeding in the cold waters of Barents Sea. However, with the advent of new, cheaper sequencing platforms and long-read technology it is now possible to develop multiple reference genome sequences representing a broader species diversity. As a contrast to NEAC, we decided here to generate a high-quality reference genome from a male Atlantic cod captured in the Celtic sea, a region representing the southernmost extreme of the Eastern Atlantic distribution (Mieszkowska *et al.* 2009; Neat *et al.* 2014) and where cod are likely to be experiencing suboptimal summer temperatures (Neat and Righton 2007). Our gadMor_Celtic assembly was built in a stepwise process involving: (i) the testing of multiple combinations of assembly parameters to generate initial assemblies using wtdgb2 (Ruan and Li 2020); (ii) the merging of contigs from selected initial assemblies into a primary assembly using quickmerge (Chakraborty *et al.* 2016); (iii) performing multiple rounds of base error correction using Racon (Vaser *et al.* 2017) and Pilon (Walker *et al.* 2014); finally (iv) the anchoring and orientation of polished contigs into sequence files representing 23 cod chromosomes.

The two ‘best’ initial assemblies (see Materials and Methods for details), were similar with regards to their total size (bp), number of contigs, and contig N50 (see Table 1), and their BUSCO scores of 20–40% indicating a poor content of identifiable reference genes. This last observation likely reflects the fact that they were constructed from nanopore reads alone (which suffer from relatively high rates of substitution and deletion errors; *e.g.*, 13% and 5% respectively (Bowden *et al.* 2019)) and that the assemblies generated were not yet corrected with higher quality reads such as those that can be generated from Illumina sequencing (Jain *et al.* 2018). To improve assembly contiguity, contigs showing a sequence overlap of more than 5kb with >95% similarities were combined using quickmerge. This increased the contig N50 from 6 to 10.4Mb and concurrently reduced the number of contigs. Thereafter, two rounds of error correction were performed. First round used Racon to generate consensus sequences using the 70X nanopore data alone and resulted in a BUSCO score of 66.5%. Second round used Pilon and 50X coverage high-quality Illumina data (16.5Mb paired-end 250 bp reads) and saw the BUSCO genome completeness score increase to 94.2% which is comparable to other high quality fish genomes (*e.g.*, *Carassius auratus* (Chen *et al.* 2019) and *Danionella translucida* (Kadobianskyi *et al.* 2019)). The resulting gadMor_Celtic assembly is composed of 1,253 contigs (contig N50 = 10.5 Mb, average contig length 0.55 Mb) and includes 686 Mb of sequence.

**Table 1:** Assembly statistics

	Total size (bp)	Total number of contigs	Contig N50 (bp)	BUSCO annotation
Wtdgb2 “Trial c”	668,357,526	1600	6,012,173	C:23.2%[D:0.3%], F:11.8%,M:65%, n = 4584
Wtdgb2 “Trial q”	670,278,278	1666	6,004,590	C:42.5%[D:0.4%], F:7.6%, M:50%, n = 4584
Quickmerge contigs	677,547,349	1253	10,448,158	Not done
Racon polishing	683,672,734	1253	10,518,163	C:66.5%[D:1.2%], F:8.1%, M:25.3%, n = 4584
**Pilon polishing**	**685,982,295**	**1253**	**10,559,872**	**C:94.1%[D:3.0%], F:1.9%, M:4.0%, n = 4584**

Metrics describing genome statistics of the two initial Wtdgb2 assemblies, the quickmerge assembly, the gadMor_Celtic assembly after polishing with nanopore (Racon) and final assembly after polishing with Illumina (Pilon) data.

High-quality linkage maps of densely spaced markers provide the means to reliably order and orient genomic fragments (contigs and scaffolds) together into sequence files representing chromosomes. If constructed in a large pedigree, and with an adequate number of markers, it may also serve as the backbone for ordering, orienting and concatenating the fragments into chromosome sequences. However, the ability to order and orientate fragments is constrained by the frequency and location of recombination events and thus is limited by the resolution of the map. In this study we used a genetic map consisting of 9,178 SNPs (Table S1), constructed in a large pedigree of 2,951 individuals to order and orientate 149 contigs (totalling 643.4 Mb; 93% of assembly) into 23 chromosome sequences. The average number of SNPs per contig was 56.1, with only 12 contigs containing fewer than five SNPs. The high contiguity of the gadMor_Celtic assembly is evidenced by the fact that for one linkage group (LG14), the entire genetic map was correctly captured by a single contig of more than 30 Mb. The total length of the female linkage map (1,662.7 cM) was approximately 1.3 times larger than the male map (1,262.3 cM). The numbers are in rough agreement with a low resolution SNP/microsatellite genetic map reporting a combined male and female map size of 1225cM and average female:male ratio of 1.78 ± 1.62 (Moen *et al.* 2009). Striking heterochiasmy is not unprecedented in teleosts, in Atlantic salmon these differences are associated with uneven distribution of recombination events, more precisely abundant male recombination at the telomers and low recombination observed elsewhere in the chromosome (Gonen *et al.* 2014).The linkage maps were constructed using genotypes from pedigreed samples belonging to families where the large inversions on LGs 1, 2, 7 and 12 (as defined by (Hubert *et al.* 2010)) were segregating, this led to pronounced gaps in the linkage maps at the boarders of these inversions (see Figure S1).

### Chromosomal inversions

**Figure 1:**
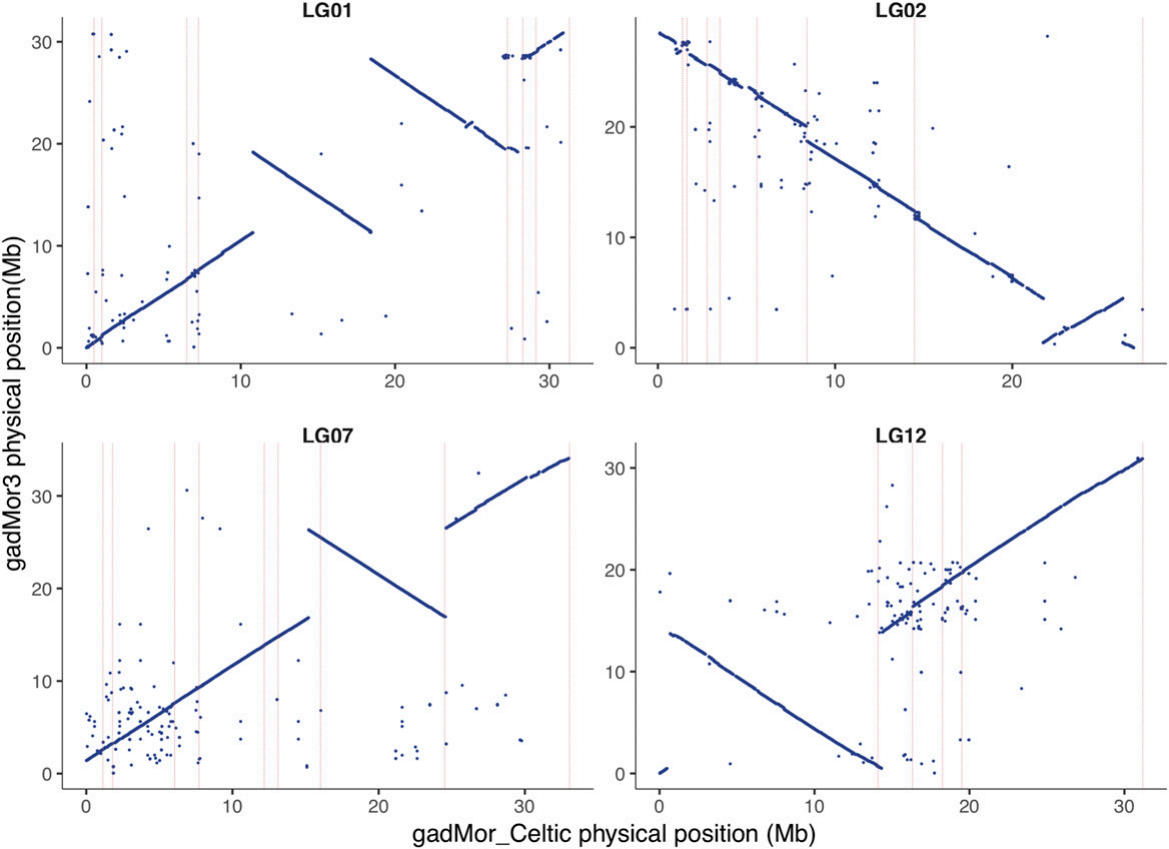
Alignment of gadMor_Celtic (x-axis) and gadMor3 (y-axis) chromosome sequences for linkage groups 1, 2, 7 and 12. Vertical lines (pink) demarcate boundaries of gadMor_Celtic contigs.

The detection of extended blocks of LD between SNPs has been used in several studies to define the regions of genetic differentiation on Atlantic cod LGs 1 2, 7 and 12 (Bradbury *et al.* 2010; Berg *et al.* 2015; Sodeland *et al.* 2016; Barney *et al.* 2017). Large chromosomal inversions have been hypothesized for all four regions but only documented for LG01 (Kirubakaran *et al.* 2016). While regions of extended LD are symptomatic of large polymorphic inversions, no studies until now have directly compared reference genomes from different cod ecotypes to define and confirm the underlying mechanism, or to locate the genomic regions containing the inversion breakpoints or to define the exact complement of genes they contain. We aligned the recently released gadMor3 assembly (NCBI accession ID: GCF_902167405.1) constructed from a NEAC individual to our gadMor_Celtic assembly using LASTZ (Harris 2007). The gadMor3 assembly was generated following a comprehensive sequencing effort combining long-read sequence data from Pacific BioSciences with various datasets for scaffolding and polishing, and resulted in 1,442 contigs (contig N50 = 1.015 Mb). Despite being an order of magnitude smaller than our gadMor_Celtic contigs, the gadMor3 scaffolds nevertheless mapped with a high confidence to the assembly and showed that the two assemblies display alternative configurations of inversions for the supergenes on LGs 1, 2, 7 and 12. In most cases, the inversion breakpoints could be described at high resolution because they locate within single nanopore contigs. Exceptions to this were the third breakpoint of LG01 and second breakpoint on LG07 which falls between two gadMor_Celtic contigs (Figure 1).

A perfect characterization of inversion breakpoints at the sequence level using the gadMor3 and gadMor_Celtic assemblies would require that contigs from both assemblies span the breakpoints and that sequences at the breakpoints would align perfectly with high confidence. As the order of contigs (relative positions) is not available in the gadMor3 assembly, and genome alignments to some extent were confounded by repetitive sequences, we believe it is appropriate to present the inversion breakpoints as regions, or putative intervals (see Table 2).

**Table 2:** Genomic regions likely containing the inversion breakpoints

Linkage Group	Putative interval containing breakpoint		Size (bp)	Inversion size (Mb)
	Start	End		
**LG01**	10,782,691	10,787,755	5,064	17.45
	18,422,802	18,425,099	2,297	
	28,225,372	28,228,130	2,758	
**LG02**	21,733,338	21,733,998	660	4.51
	26,233,253	26,238,098	4,840	
**LG07**	15,208,043	15,210,043	2,000	9.37
	24,574,346	24,575,510	1,164	
**LG12**	493,527	635,659	142,132	13.88
	14,330,965	14,376,973	46,008	

A pairwise comparison between gadMor_Celtic and gadMor3 reveals the interval (start and stop coordinates relative to the gadMor_Celtic assembly) for each inversion breakpoint in LGs 1, 2, 7, and 12.

Localizing precise inversion breakpoints can be informative in functional studies as they may directly impact gene structure, such as has been reported in cattle (Escouflaire *et al.* 2019) or gene expression; by disrupting regulatory elements. Similarly, analyzing sequences close to a breakpoint may highlight signatures accompanying inversions such as the inverted repeat reported in Atlantic Herring (Pettersson *et al.* 2019) and provide insight into mechanisms by which inversions arise. Finally, a major effect of inversions in an evolutionary context is the inhibition of recombination and protection of linked favorable alleles (Kirkpatrick 2010) implying that localizing the precise boundaries of inversions enables cataloguing which genes they contain and the genetic differences that define the inversion haplotype.

In the gadMor_Celtic assembly the double inversion on LG01 spans a total interval of 17.45 Mb which is slightly larger than our previous estimate of 17.37 Mb (Kirubakaran *et al.* 2016). Our ability to detect inversions when comparing gadMor3 to the NEAC reference suggests that Celtic cod possess the stationary (as opposed to migratory) ecotype chromosome configuration. An earlier survey of Celtic cod (Neat *et al.* 2014) showed that while a portion of the population migrate a significant distance from the Celtic sea to the Western English channel they do not attempt to swim at depth, unlike the migratory NEAC fish which have been found at depths of up to 500m (Godø and Michalsen 2000). Celtic cod are typically located at depths of about 100 meters, which is similar to the depth distribution of the stationary populations found around the Norwegian coast (Hobson *et al.* 2007).

The inversions on LGs 2, 7 and 12 span 4.51, 9.37 and 13.88 Mb, respectively. These sizes are in relatively close agreement to earlier estimations of 5.0, 9.5, and 13 Mb, which were calculated from LD analyses and detection of regions of elevated divergence between populations (Sodeland *et al.* 2016). In their analysis, Sodeland *et al.* (2016) used the highly fragmented gadMor1 assembly (Star *et al.* 2011) and a relatively sparse set of 9,187 SNPs to define the regions, both factors that may explain the physical difference between estimates. A more recent study investigated cod populations from the Northwest Atlantic and measured LD among almost 3.4M SNPs detected from resequencing data, the LGs 2, 7 and 12 inversions were estimated to be 5.6, 9.3, and 11.6 Mb respectively (Barney *et al.* 2017). While not identical, these regions and sizes detected in fish from both sides of the Atlantic are remarkably consistent, supporting the hypothesis that these cod have a common ancestral origin (Berg *et al.* 2017; Sinclair-Waters *et al.* 2017).

**Figure 2:**
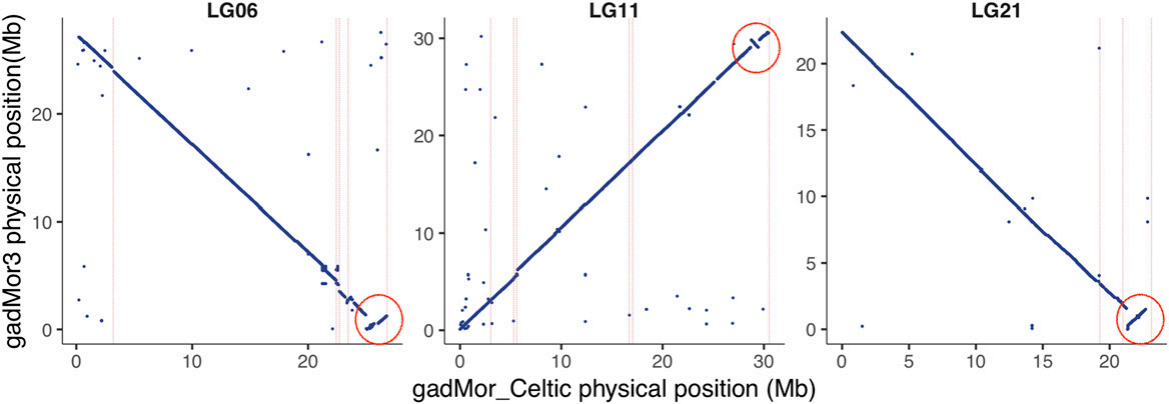
Putative inversions detected on LGs 6, 11 and 21.

Our analyses suggest the presence of putative inversions in gadMor_Celtic on LGs 6, 11 and 21 (see Figure 2) which, to the best of our knowledge, have not been reported elsewhere. The inversions are smaller (1.4, 0.6, 1.78 Mb, respectively) than the rearrangements comprising the supergenes on LGs 1, 2, 7 and 12.

### Annotation of gene content and repetitive elements

There is a growing body of evidence that chromosomal inversions in fishes can capture multiple adaptive alleles and therefore act as supergenes (for example (Jones *et al.* 2012; Pearse *et al.* 2019; Pettersson *et al.* 2019). Defining the gene content and identifying genetic variation within these chromosomal inversions is an important means for investigating how changes in genome organization may lead to phenotypic and adaptive divergence. Utilizing available transcript data we predict 14,292 genome wide gene models with 735, 236, 343 and 452 gene models predicted in inversions on LGs 1, 2, 7 and 12 respectively (Table S3). To document repeats in gadMor_Celtic we created a repeat library using RepeatModeler (Smit *et al.* 1996) which, when used with RepeatMasker (Smit *et al.* 1996) saw almost one third of the genome (32.26%) classified as repetitive.

### Potential centromere structure and organization

**Figure 3:**
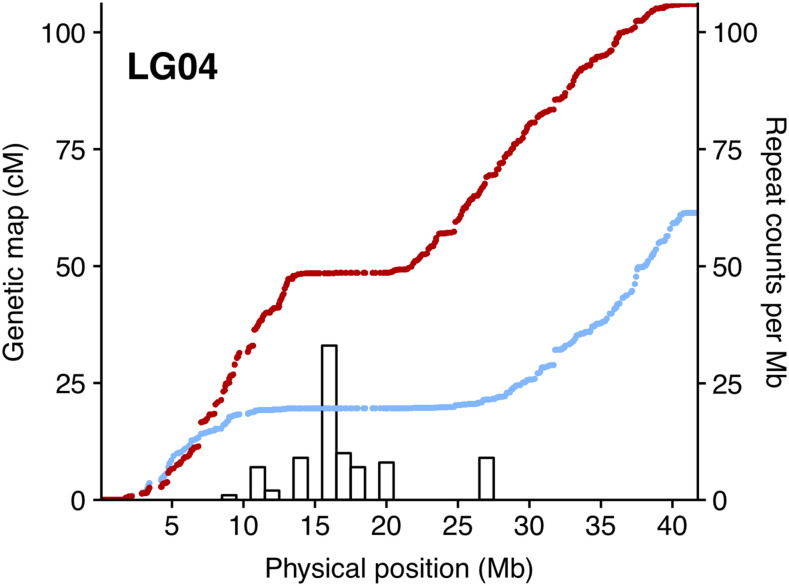
Position of potential centromere related sequence on LG04. Collinearity between LG04 genetic maps for males (red) and female (blue) and the frequency of a 258bp tandem repeat structure (histogram) predicted to be related to centromeres.

Centromeres contribute to the physical linking of sister chromatids during meiosis and their location within a dyad is important for defining the chromosomal morphology (or chromosome classification) used in karyotyping studies (*e.g.*, metacentic, acrocentric, etc). Centromeres can be relatively large and usually contain a lot of repetitive, but poorly conserved sequences (Melters *et al.* 2013). Searching for known centromere repeats (Melters *et al.* 2013) in gadMor_Celtic assembly failed to reveal any convincing hits. We therefore used TandemRepeat finder (TRF) (Benson 1999) to scan the assembly for seqences meeting characteristics typical of centromeric repeats; specifically containing more than 60% AT, longer than 80 bp, and present in all 23 LGs. We detected a 258bp sequence composed of two identical and similarly oriented 88bp repeats (one at each end) separated by an 82bp interveining sequence (see File S2 for details). This expected centromeric repeat appeared 806 times (with more than 95% identity) across the genome and was found on all LGs. The location of this repeat was compared to the genetic map profiles for all 23 linkage groups (Figure S1). We reasoned that regions of reduced recombination likely contain, or are close to, the centromere and should therefore coincide with the mapping of the centromeric repeat sequence. For most linkage groups, there was a convincing overlap between these two metrics. Most evidently, all four LGs (2, 4, 10 and 12) showing clear sigmoidal linkage profiles characteristic of a metacentric chromosome (Ghigliotti *et al.* 2012), contained expansion of the centromeric repeat sequence within the region of repressed recombination in the middle of the linkage group (see Figure 3 for example). It should be noted however that reduced recombination is also a feature of inversions meaning that both centromers and inversions help to shape recombination profiles.

In this paper we used nanopore sequencing to generate a chromosome-level genome assembly from a male Atlantic cod captured in the Celtic Sea. Cod from this region experience high, possibly suboptimal summer temperatures, and consequently this sample represents a contrast to the current genome assemblies generated from NEAC population sampled from the considerably colder Barents Sea. By generating this new assembly, and comparing it against the gadMor3 assembly, we were able to characterize the population specific chromosomal rearrangements associated with four notable supergenes displaying pronounced divergence between them. Pairwise comparison of the two genomes also revealed additional putative rearrangements on LGs 6, 11 and 21, which has not been reported before. Identification and mapping of the centromeric repeat enabled by the new high resolution gadMor_Celtic assembly, combined with linkage maps, were used to study chromosomal morphology and reliably identify four characteristic metacentric chromosomes in Atlantic cod.
